# Efficacy and Safety of Non-recommended Dose of New Oral Anticoagulants in Patients With Atrial Fibrillation: A Systematic Review and Meta-Analysis

**DOI:** 10.3389/fcvm.2021.774109

**Published:** 2021-12-23

**Authors:** Xiangyun Kong, Yong Zhu, Lianmei Pu, Shuai Meng, Lihan Zhao, Wei Zeng, Weiyan Sun, Guangming Wu, Hong Li

**Affiliations:** ^1^Department of General Medicine, Beijing Luhe Hospital, Capital Medical University, Beijing, China; ^2^Department of Cardiology, Beijing Anzhen Hospital, Capital Medical University, Beijing, China; ^3^Department of Cardiology and Macrovascular Disease, Beijing Tiantan Hospital, Capital Medical University, Beijing, China

**Keywords:** atrial fibrillation, new oral anticoagulants, non-recommended dose, bleeding, stroke, meta-analysis

## Abstract

**Introduction:** The real-world treatment of atrial fibrillation (AF) often involves the prescription of new oral anticoagulants (NOACs) using dosing both lower and higher than recommended guidelines. Our study aimed to evaluate the efficacy and safety of non-recommended dosage of NOACs in AF patients.

**Methods:** A systematic search was performed for relevant studies across multiple electronic databases (PubMed, Embase, Cochrane Library, Clinical Trials Registry) from inception to May 1, 2021. Multicenter randomized trials and observational studies were selected with key reporting measures for inclusion involved efficacy outcomes including stroke or systemic thromboembolism along with safety endpoints assessing major or clinically relevant bleeding events.

**Results:** A total of 11 eligible studies were included involving 48,648 patients receiving recommended dose of NOACs and 50,116 patients receiving non-recommended dosage. Compared to AF patients treated with recommended dose regimens, administration of low dose of NOACs was associated with higher risk of stroke/systemic embolism (RR = 1.24, 95% CI 1.14–1.35, *P* < 0.00001), but without reducing bleeding risk (RR = 1.18, 95% CI 0.91–1.53, *P* = 0.21) and a higher risk of all-cause mortality (RR = 1.58, 95% CI 1.25–1.99, *P* = 0.0001). Moreover, high dose of NOACs was associated with higher risk of stroke and systemic embolism efficacy (RR = 1.71, 95% CI 1.06–2.76, *P* = 0.03) and a non-significant trend to a greater risk of major or clinically relevant bleeding (RR = 1.57, 95% CI 0.96–2.58, *P* = 0.07).

**Conclusions:** AF patients treated with low dose of NOACs showed equivalent safety but with worse efficacy compared with recommended dose. High dose of NOACs was not superior to recommended dose regimens in preventing stroke/systemic embolism outcomes in AF patients.

## Introduction

The past two decades have witnessed the gradual implementation of four new oral anticoagulants (dabigatran, rivaroxaban, apixaban, edoxaban) to prevent stroke in patients with non-valvular AF. These agents have shown greater efficacy and safety compared with vitamin K antagonists (VKAs) (1) and have been widely approved by regulatory bodies including the European Medicines Agency (EMA) (2), the U.S. Food and Drug Administration (FDA) (3), and the Japanese Pharmaceuticals and Medical Devices Agency (PMDA) (4). Nevertheless, in real-world clinical practice, safety concerns among physicians have led to prescribing habits of lower dose of NOACs in patients with high HAS-BLED scores, along with considerations of age, body mass index (BMI), creatinine clearance (CrCl), hepatic function, as well as concomitant disease states (5). Alternatively, patients with significantly high CHA2DS2-VASc scores might be given higher dose of NOACs (6). These scenarios provide concerns that the efficacy and safety of NOACs may be compromised. At present, there are no universal rules for determining the dose regimens in high-risk patients, and no clear risk-benefit analysis was performed to help to adjust NOAC dose regimens. In this study, we performed a meta-analysis of multicenter randomized trials and observational studies to evaluate the efficacy and safety of non-recommended dose compared with recommended dose of NOACs in patients with AF.

## Materials and Methods

This systematic review and meta-analysis were performed following the recommendations of the Cochrane Handbook for Systematic Reviews of Interventions and Preferred Reporting Items for Systematic Reviews and Meta-Analysis statement (PRISMA).

### Search Strategy

Two investigators independently performed comprehensive searches for all relevant articles against the following databases: PubMed, Embase, Cochrane Library databases and Clinical Trials Registry (www.clinicaltrials.gov). English language articles were searched from inception to May 1, 2021. The search terms keywords used were as follows: atrial fibrillation; new oral anticoagulants, non-vitamin K antagonist, NOAC, oral thrombin inhibitors, factor Xa inhibitors, apixaban, rivaroxaban, edoxaban, dabigatran; non-recommended dose, off-label dose, low-dose, reduced-dose, underdosing, high dose, overdose; stroke; bleeding. Additionally, the reference lists of related review articles were also reviewed to source additional publications of relevance.

### Inclusion and Exclusion Criteria

Manually selected studies with the assistance of EndNote software according to the prespecified PICOS criteria. Studies that met the following criteria were selected for inclusion involved: (1) P: patients with atrial fibrillation; (2) Intervention: treated with non-recommended dose (low or high dose) of new oral anticoagulants (NOACs: dabigatran, rivaroxaban, apixaban, edoxaban); (3) Comparison: with recommended dose of NOACs; (4) Outcomes: reported at least one of the following adverse outcomes: stroke/systemic embolism, major bleeding, all-cause mortality, cardiovascular cause of death by dose subgroups; (5) Study design: RCTs and observational studies published as full articles.

The exclusion criteria were as follows: (1) Compared anticoagulation strategies using NOACs vs. warfarin; (2) Other types of studies, including case reports, animal experiments, meta-analysis, reviews, comments, editorials, and conference abstracts; (3) Reported exposure or endpoints not suitable for our analysis.

### Data Extraction and Quality Assessment

Two investigators independently extracted data from the eligible studies. Baseline characteristics of the patients (age, sex, creatinine clearance, BMI, CHADS2/CHA2DS2-VASc score, HAS-BLED score), study design, follow-up periods and the prespecified adverse outcomes were retrieved. In addition, they also assessed the quality of the RCTs and observational studies using the Revised Jadad's Scale and Newcastle-Ottawa Scale (NOS), respectively. Any disagreements or uncertainties between the two reviewers in the processes of study selection, data extraction and quality assessment were resolved by discussion with the senior investigator (HL).

### Definitions and Outcomes

The criteria of standard dose approved by guidelines lately as well as the definitions of recommended/low/high dose of NOACs in our selected studies are listed in Table 1 (5, 7–19). The primary efficacy outcomes were stroke or systemic embolism (SE) and the primary safety outcomes were major or clinically relevant bleeding. For trials reporting only major bleeding, the same data were used for major or clinically relevant bleeding. The definition of major bleeding was in accordance with the International Society on Thrombosis and Hemostasis (ISTH) criteria (10, 20). The secondary outcomes included all-cause mortality, cardiovascular cause of death, if reported data were available.

**Table 1:** The definitions of recommended/low/high NOACs dosage.

**NOAC**	Standard dose^*^	**Recommended dose^#^**	**Low dose**	**High dose**
Dabigatran	Standard dose: 150 mg bid; Dose-reduction criteria: 110 mg bid in patients with: (1) Age ≥80 years; (2) Increased bleeding risk; (3) Concomitant use of verapamil (7).	Recommended dose:150 mg bid; 1. Dose-reduction criteria (11, 13): 110 mg bid if any of 3 criteria was met: (1) Age ≥80 y; (2) Age 75–80 y with high risk of bleeding; (3) Concomitant use of verapamil. 2. Dose-reduction criteria (12): 110 mg bid if any of 3 criteria was met: (1) Age ≥70 years; (2) CrCl: 30–50 mL/min; (3) Prior gastro-intestinal bleeding, or concomitant use of oral P-glycoprotein inhibitors. 3. Dose-reduction criteria (13): 75 mg bid in patients with: (1) CrCl: 15–30 mL/min (2) Concomitant dronedarone.	110 mg bid for patients without any dosage reduction criteria.	Dabigatran 150 mg bid if any dosage reduction criteria were met OR Use of dabigatran if CrCl <30 mL/min.
Rivaroxaban	Standard dose: 20 mg QD; Dose-reduction criteria: 15 mg QD if CrCl ≤ 15–49 mL/min (7, 8).	Recommended dose (ROCKET AF): 20 mg QD Dose-reduction criteria: 15 mg QD if CrCl <50 mL/min (5, 9–11). Recommended dose (J-ROCKET AF): 15 mg QD Dose-reduction criteria: 10 mg QD if CrCl <50 mL/min (12).	Rivaroxaban 15 mg/10 mg QD if CrCl ≥50 mL/min	Rivaroxaban 20 mg QD if CrCl <50 mL/min OR use of rivaroxaban if CrCl <15 mL/min.
Apixaban	Standard dose: 5 mg bid; Dose-reduction criteria:2.5 mg bid if 2 out of 3 fulfilled: (1) Age ≥80 years; (2) Weight ≤ 60 kg; (3) Serum creatinine ≥133 mmol/L (1.5 mg/dL) (OR single criterion: if CrCl 15–29 mL/min) (7, 8).	Recommended dose:5 mg bid; Dose-reduction criteria: 2.5 mg bid if ≥2 of 3 criteria were met: (1) Age ≥80 y; (2) Body weight ≤ 60 kg; (3) Serum creatinine ≥1.5 mg/dL (OR single criterion: if CrCl 15–30 mL/min) (10).	Apixaban 2.5 mg bid if dosage reduction criteria were not met	Apixaban 5 mg bid for patients who met the dosage reduction criteria OR use of apixaban if CrCl <15 mL/min.
Edoxaban	Standard dose: 60 mg QD; Dose-reduction criteria: 30 mg QD if any of 3 criteria was met: (1) Body weight ≤ 60 kg; (2) CrCl 30–50 mL/min; (3) Concomitant use of verapamil, quinidine, or dronedarone (7).	Recommended dose:60 mg QD; Dose-reduction criteria: 30 mg QD if any of 3 criteria was met: (1) Body weight ≤ 60 kg; (2) CrCl <50 mL/min; (3) Use of P-glycoprotein inhibitor (13–15).	30 mg QD for patients who did not meet the dosage reduction criteria OR use of edoxaban 15 mg QD.	60 mg QD for patients who met the dosage reduction criteria OR use of edoxaban if CrCl <15 mL/min.

* *Standard dose approved by guidelines*;

# *Recommended dose of selected studies. CrCl, creatinine clearance*.

### Statistical Analysis

Comparison of the treatment effects of non-recommended dose of NOACs (low/high dose) vs. recommended dose of NOACs was performed used risk ratios (number of events or the incidence in each treatment group) and respective 95% confidence intervals. The heterogeneity across the studies was assessed by Cochran's *Q*-test (*P* < 0.1 was regarded as statistically significant) and *I*^2^ statistics, which estimate heterogeneity quantitatively (*I*^2^ value < 25% indicates no or mild heterogeneity, *I*^2^ > 75% indicates high heterogeneity). If *I*^2^ ≤ 50% and *P* ≥ 0.1, the fixed-effects model was used. If *I*^2^ > 50% or *P* < 0.1, data were pooled used random-effects, according to the Mantel-Haenszel model, and the cause of heterogeneity was sought. Publication bias assessment was made through visual inspection of the asymmetry in funnel plots. All statistical analyses were performed using Review Manager software (Rev- Man) version 5.3 (Cochrane Collaboration 2014, Nordic Cochrane Center, Copenhagen, Denmark). Two-tailed *P*-values < 0.05 were considered significant.

## Results

### Study Selection and Characteristics of the Eligible Studies

**Figure 1:**
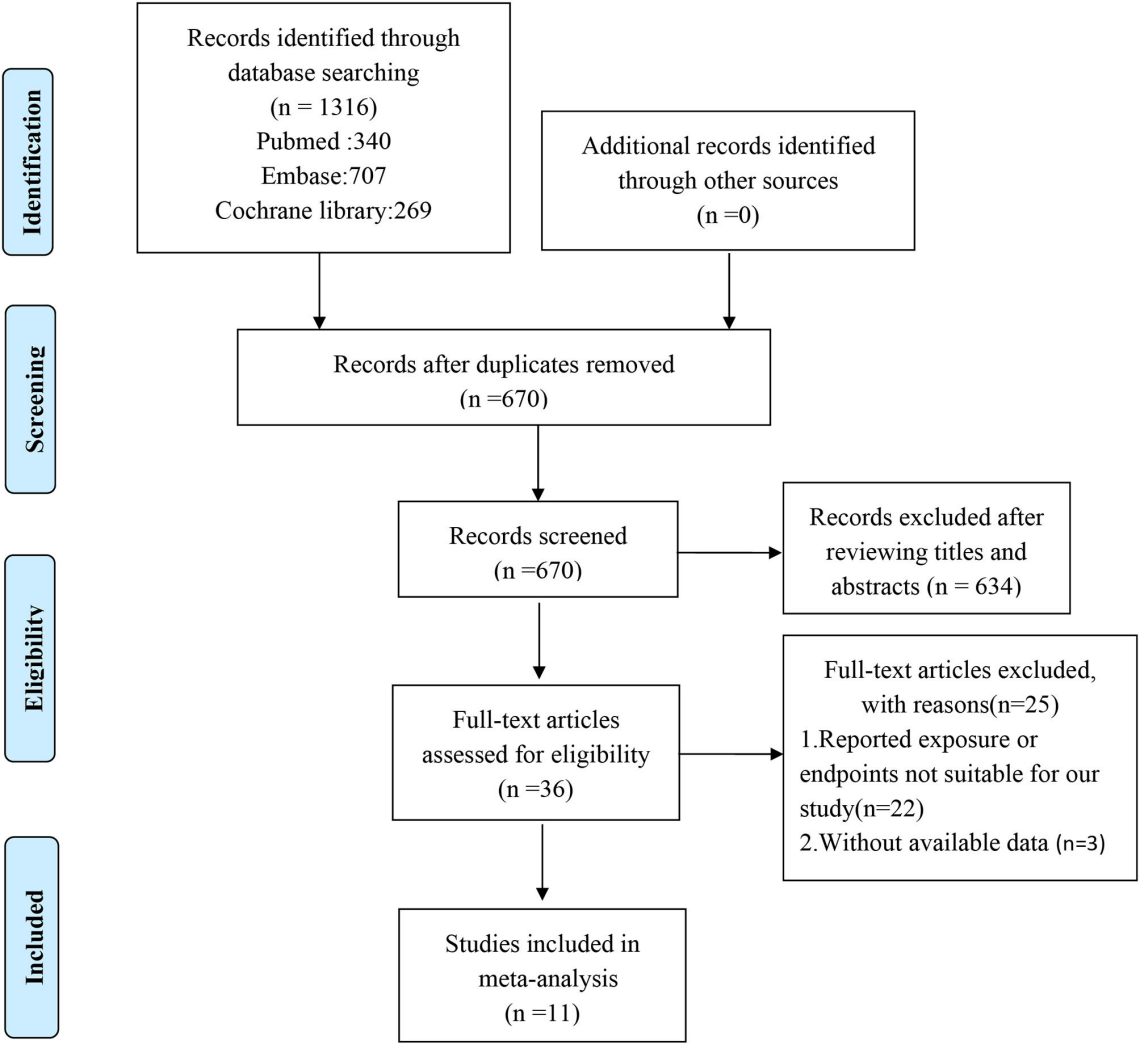
The flow chart of study selection.

Our search strategy identified 1,316 potentially relevant studies, of which 670 records were retrieved after duplicates were removed. After assessing titles and abstracts, a total of 36 full-text articles were eligible for further screening. Of these, 25 records were excluded for not meeting the eligibility criteria, leaving 11 articles for further consideration (5, 9–15, 17–19). The study selection flowchart is presented in Figure 1.

Overall assessment of the 11 eligible studies showed data for 100,131 total patients to be included in the meta-analysis. Of these, 48,648 received recommended dose of NOACs whereas 50,116 received non-recommended dose of NOACs. Among the latter group, 49,384 patients were prescribed lower than recommended dose while 732 patients were given higher dose. Articles were published from 2011 to 2021, and most of them were non-randomized, observational and comparative studies. Importantly, the 11 identified trials were rated good for methodological quality according to assessment by NOS and Revised Jadad's scales. The general features of the 11 studies are summarized in Table 2 with corresponding reported endpoints and follow-up periods listed in Table 3.

**Table 2:** General features of the eligible studies.

**References**	**Study type**	**Intervention (*n*)**	**Recommended dose (*n*)**	**Low dose (*n*)**	**High dose (*n*)**	**Study quality**
Benjamin et al. (10)	OC	4 DOACs (*n* = 7,925)	6,376	555	NA	8
Camm et al. (11)	OC	4 DOACs (*n* = 10,426)	7,603	2,423	400	8
Cho et al. (12)	OC	3 NOACs (*n* = 46,095): Dabigatran (*n* = 12,593) Rivaroxaban (*n* = 21,000) Apixaban (*n* = 12,502)	Dabigatran (*n* = 3,138) Rivaroxaban (*n* = 8,601) Apixaban (*n* = 4,661)	Dabigatran (*n* = 9,455) Rivaroxaban (*n* = 12,399) Apixaban (*n* = 7,841)	NA	7
Chung et al. (13)	RCT	Edoxaban (*n* = 159)	80	79	NA	5
Ezekowitz et al. (18)	OC	Dabigatran (*n* = 5,851)	2,937	2,914	NA	8
Fernández et al. (9)	OC	Rivaroxaban (*n* = 1,421)	1,183	138	100	8
Huisman et al. (19)	OC	Dabigatran (*n* = 2,937)	1,748	1,106	NA	8
Inoue et al. (17)	OC	Dabigatran (*n* = 6,443)	1,571	4,759	NA	8
Pierre et al. (5)	OC	Rivaroxaban (*n* = 4,464)	3,608	583	232	8
Steffel et al. (15)	RCT	Edoxaban (*n* = 14,014)	7,012	7,002	NA	6
Yamashita et al. (14)	RCT	Edoxaban (*n* = 396)	130	130	NA	5

*OC, observational cohorts; RCT, randomized controlled trials; NOACs, new oral anticoagulants; NA, not available*.

**Table 3:** Follow up periods and reported outcomes.

**Studies**	**Enrollment period**	**Follow-up periods**	**Efficacy outcome**	**Safety outcome**
Benjamin et al.	2013–2016	1 year	Stroke or SE or TIA	Major bleeding (ISTH)
Camm et al.	2010–2016	2 years	Stroke/SE	Major bleeding
Cho et al.	2015–2016	15 months	Thromboembolic events (ischemic stroke or SE)	Major bleeding
Chung et al.	2007–2008	3 months	All adverse event: MACE, consisting of stroke (ischemic or hemorrhagic), SE, MI, CV death and hospitalization for any other cardiac condition	All bleeding events (major, clinically relevant non-major and minor)
Ezekowitz et al.	2005–2012	4.6 years	Stroke (ischemic, hemorrhagic, or unspecified), SE, MI, hospitalization, vascular mortality, and total mortality	Major, life threatening, GI, Intracranial, extra-cranial, minor, and fatal bleeding
Fernández et al.	NA	2.5 years	Thromboembolic events (stroke, TIA, SE or MI)	Major bleeding (ISTH)
Huisman et al.	NA	24 months	Stroke (ischemic or hemorrhagic)	Major bleeding
Inoue et al.	2011–2013	610 days	Stroke, TIA, SE	Any bleeding
Pierre et al.	2013–2014	1 year	Thromboembolic events (stroke, TIA, non-CNS SE, or MI)	Major bleeding (ISTH)
Steffel et al.	NA	2.8 years	Stroke/systemic embolism (SE)	Major bleeding (ISTH)
Yamashita et al.	2007–2008	8 weeks	Thromboembolic events	All bleeding events (major, clinically relevant non-major, and minor bleeds)

*NA, not available; SE, systemic embolism; MI, myocardial infarction; TIA, transient ischemic attack; CNS, central nervous system; CV, cardiovascular; GI, gastrointestinal; ISTH, International Society of Thrombosis and Hemostasis; MACE, major adverse cardiovascular events*.

### Baseline Characteristics of Patients Enrolled in the Eligible Studies

The baseline demographic characteristics of AF patients treated with low/high dose of NOACs vs. recommended dose are presented in Tables 4, 5, respectively. The patients receiving non-recommended dose tended to be elderly, more likely be female, have low body weight, and show high HAS-BLED or CHA2DS2-VASc scores.

**Table 4:** Baseline characteristics of recommended dose and non-recommended low dose of NOACs.

**Studies**	**Age, M ± SD/(IQR) RD/LD**	**Male, *n* (%) RD/LD**	**BMI, M ± SD/(IQR) RD/LD**	**CrCl, M ± SD/(IQR) RD/LD**	**HAS-BLED score, M ± SD/(IQR) RD/LD**	**CHADS2 score, M±SD RD/LD**	**CHA2DS2-VASc score, M±SD/(IQR), RD/LD**
Benjamin et al.	69.0 (62.0, 75.0)/ 84.0 (81.0, 88.0)	3975 (62.3%)/ 202 (36.4%)	NA	NA	NA	NA	NA
Camm et al.	70.0 (63.0, 77.0)/ 77.0 (69.0, 83.0)	4,435 (58.3%)/ 1,192 (49.2%)	27.4 (24.3, 31.2)/ 25.3 (22.8, 28.8)	NA	1.0 (1.0, 2.0)/ 1.0 (1.0, 2.0)	NA	3.0 (2.0, 4.0)/ 4.0 (3.0, 5.0)
Cho et al.	NA	NA	NA	NA	NA	NA	NA
Chung et al.	65.9 ± 7.7/ 64.9 ± 9.1	55 (68.8%)/ 51 (64.6%)	NA	NA	NA	1.9 ± 1.0/ 2.0 ± 1.1	3.1 ± 1.4/ 3.2 ± 1.4
Ezekowitz et al.	NA	NA	NA	NA	NA	NA	NA
Fernández et al.	73.0 ± 9.6/ 78.4 ± 8.7	673 (56.9%)/ 74 (53.6%)	NA	NA	1.5 ± 1.0/ 2.0 ± 1.0	1.9 ± 1.2/ 2.4 ± 1.4	3.4 ± 1.5/ 4.0 ± 1.7
Huisman et al.	67.2 ± 9.6/ 74.6 ± 9.3	1,049 (60.0%)/ 544 (49.2%)	30.3 ± 6.4/ 27.5 ± 5.0	93.9 ± 37.3/ 68.4 ± 26.6	1.1 ± 0.9/ 1.4 ± 0.8	1.7 ± 1.0/ 2.2 ± 1.1	2.9 ± 1.3/ 3.7 ± 1.4
Inoue et al.	63.1 ± 9.1/ 73.3 ± 8.7	1,259 (80.1%)/ 2,990 (62.8%)	24.7 ± 3.7/ 23.8 ± 3.5	90.0 ± 27.7/ 67.6 ± 23.0	1.5 ± 1.0/ 2.3 ± 1.1	1.4 ± 1.1/ 2.0 ± 1.3	2.1 ± 1.5/ 3.3 ± 1.6
Pierre et al.	70.5 ± 9.9/ 76.7 ± 8.9	60.7%/55.2%	28.4 ± 5.0/ 27.6± 4.6	NA	2.0 ± 1.0/ 2.3 ± 1.1	1.9 ± 1.3/ 2.5 ± 1.2	3.3 ± 1.7/ 4.1 ± 1.5
Steffel et al.	72.0 (64.0, 78.0)/ 72.0 (64.0, 78.0)	4,353 (62.1%)/ 4,284 (61.2%)	NA	70.4 (53.8, 92.4)/ 70.3 (53.8, 92.2)	NA	NA	4 (3.0, 5.0)/ 4 (3.0, 5.0)
Yamashita et al.	68.4/ 69.4	107/ 110	24.7/ 24.6	NA	NA	2.1/ 1.9	NA

*M ± SD, mean ± standard deviation (IQR interquartile range); RD, recommended dose; LD, low dose; NA, not available; BMI, body mass index; CrCl, creatinine clearance*.

**Table 5:** Baseline characteristics of recommended dose and non-recommended high dose of NOACs.

**Studies**	**Age, M ± SD/(IQR) RD/HD**	**Male, *n* (%) RD/HD**	**BMI, M ± SD/(IQR) RD/HD**	**CrCl, RD/HD**	**HAS-BLED score, M ± SD/(IQR) RD/HD**	**CHADS2 score, M ± SD, RD/HD**	**CHA2DS2-VASc score, M ± SD/(IQR) RD/HD**
Camm et al.	70.0 (63.0, 77.0)/ 75.0 (68.0, 82.0)	4435 (58.3%)/ 206 (51.5%)	27.4 (24.3, 31.2)/ 27.4 (23.9, 32.0)	NA	1.0 (1.0, 2.0)/ 2.0 (1.0, 2.0)	NA	3.0 (2.0, 4.0)/ 4.0 (3.0, 5.0)
Fernández et al.	73.0 ± 9.6/ 82.3 ± 5.6	673 (56.9%)/ 41 (41.0%)	NA	NA	1.5 ± 1.0/ 1.9 ± 0.9	1.9 ± 1.2/ 2.3 ± 1.1	3.4 ± 1.5/ 4.2 ± 1.3
Pierre et al.	70.5 ± 9.9/ 76.3 ±8.0	60.7%/ 40.1%	28.4 ± 5.0/ 26.8 ± 5.4	NA	2.0 ± 1.0/ 2.4 ± 1.0	1.9 ± 1.3/ 2.5 ± 1.3	3.3 ± 1.7/ 4.4 ± 1.6

*M ± SD, mean ± standard deviation (IQR interquartile range);RD, recommended dose; HD, high dose; NA, not available; BMI, body mass index; CrCl, creatinine clearance*.

### Efficacy Outcomes

**Figure 2:**
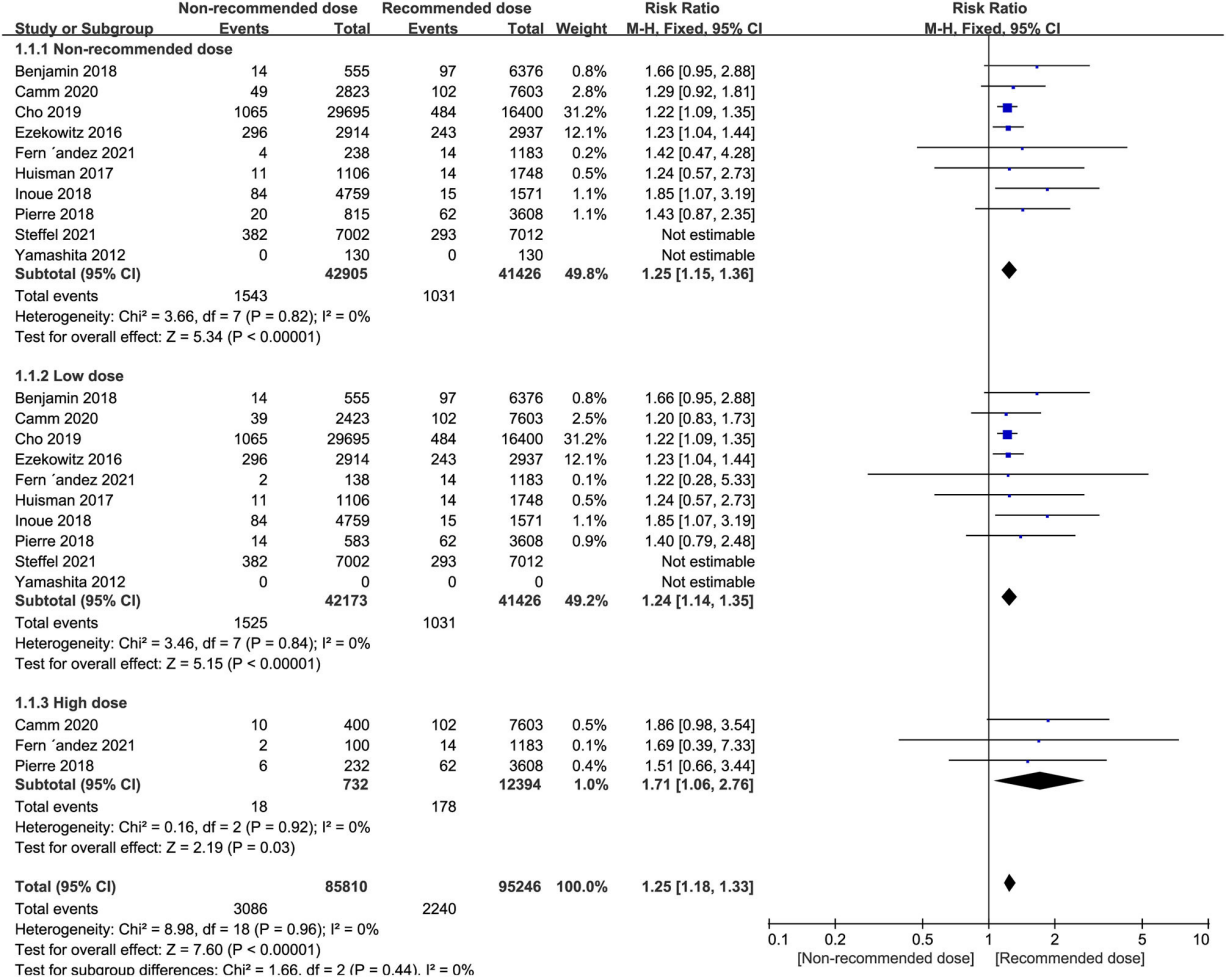
Forest plot for stroke or systemic embolism in non-recommended dose compared to recommended dose of NOACs. NOACs, new oral anticoagulants.

The results of our pooled indicated that the risk of stroke/systemic embolism in AF patients using non-recommended dose of NOACs was significantly higher than patients using the recommended dose (RR = 1.25, 95% CI 1.15–1.36, *P* < 0.00001). Moreover, similar results were also observed when either low dose (RR = 1.24, 95% CI 1.14–1.35, *P* < 0.00001) or high dose groups (RR = 1.71, 95% CI 1.06–2.76, *P* = 0.03) were compared against the recommended dose group (Figure 2).

We next conducted subgroup analyses comparing the individual NOAC agents separately between the non-recommended dose and recommend dose categories. Notably, we found that patients prescribed low dose of NOACs, no matter dabigatran, apixaban or edoxaban, showed a higher risk of stroke/systemic embolism than patients administered the recommended dose. In contrast, patients receiving non-recommended dose of rivaroxaban (low/high) were not at greater risk of stroke/systemic embolism than the standard dose (Table 6).

**Table 6:** Stroke or systemic embolism of low/high dose of individual NOACs compared to recommended dose.

**Low dose vs. RD**	**No. of studies**	**No. of participants**	**P for heterogeneity**	***I*^2^** **(%)**	**RR (95% CI)**	**P for test**
Dabigatran	4	27,628	0.37	5%	1.34 (1.18, 1.52)	<0.00001
Rivaroxaban	3	26,512	0.53	0%	1.02 (0.89, 1.18)	0.78
Apixaban	1	12,502	–	–	1.46 (1.18, 1.82)	0.0006
Edoxaban	2	14,274	–	–	1.31 (1.13, 1.51)	0.0004
**High dose vs. RD**	**No. of studies**	**No. of participants**	**P for heterogeneity**	***I*^2^** **(%)**	**RR (95% CI)**	**P for test**
Rivaroxaban	2	5,123	0.89	0%	1.55 (0.75, 3.18)	0.24

*RD, recommended dose; CI, confidence interval*.

### Safety Outcomes

**Figure 3:**
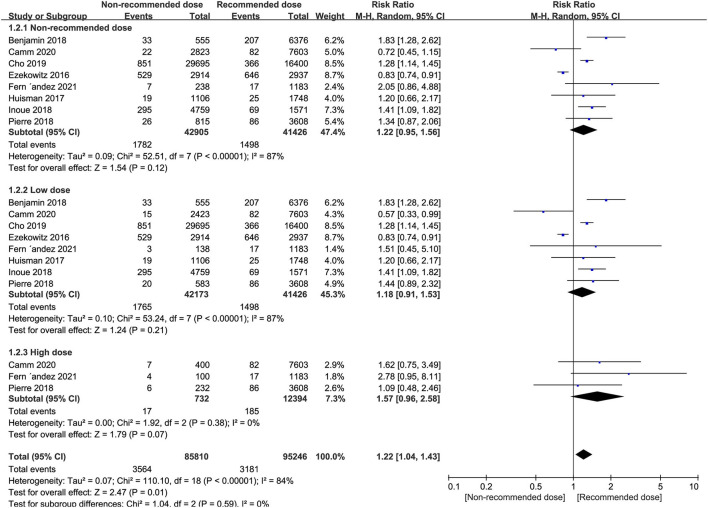
Forest plot for major or clinically relevant bleeding in non-recommended dose compared to recommended dose of NOACs. NOACs, new oral anticoagulants.

Patients administered non-recommended dose of NOACs did not experience more major or clinically relevant bleeding events than patients treated with recommended dose (RR = 1.22, 95% CI 0.95–1.56, *P* = 0.12). Similarly, equal safety outcomes were found after dividing patients into either low or high dose groups relative to patients treated with recommended dose (*I*^2^ = 87%, RR = 1.18, 95% CI 0.91–1.53, *P* = 0.21; *I*^2^ = 0%, RR = 1.57, 95% CI 0.96–2.58, *P* = 0.07, respectively) (Figure 3).

However, subgroup analyses revealed that patients receiving low dose rivaroxaban or apixaban, exhibited a significantly higher risk of bleeding compared to those on recommended dose (RR = 1.21, 95% CI 1.04–1.41, *P* = 0.01; RR = 1.42, 95% CI 1.09–1.85, *P* = 0.01, respectively). In contrast, superior safety was encountered in the low dose edoxaban group (RR = 0.67, 95% CI 0.58–0.76, *P* < 0.00001) whereas there were no safety differences found between patients receiving low and recommended dose of dabigatran (*I*^2^ = 90%, RR = 1.21, 95% CI 0.81–1.80, *P* = 0.36). Intriguingly, patients administered high dose rivaroxaban showed a non-significant trend toward increased major or clinically relevant bleeding above the standard dose (*I*^2^ = 48%, RR = 1.43, 95% CI 0.75–2.72, *P* = 0.28) (Table 7).

**Table 7:** Major or clinically relevant bleeding of low/high dose of individual NOACs compared to recommended dose.

**Low dose vs. RD**	**No. of studies**	**No. of participants**	**P for heterogeneity**	***I*^2^** **(%)**	**RR (95% CI)**	**P for test**
Dabigatran	4	2,7628	<0.00001	90%	1.21 (0.81, 1.80)	0.36^*^
Rivaroxaban	3	26,512	0.71	0%	1.21 (1.04, 1.41)	0.01
Apixaban	1	12,502	–	–	1.42 (1.09, 1.85)	0.01
Edoxaban	3	14,433	0.70	0%	0.67 (0.58, 0.76)	<0.00001
**High dose vs. RD**	**No. of studies**	**No. of participants**	**P for heterogeneity**	***I*^2^** **(%)**	**RR (95% CI)**	**P for test**
Rivaroxaban	2	5,123	0.17	48%	1.43 (0.75, 2.72)	0.28

* *Random-effects models. RD, recommended dose; CI, confidence interval*.

### All-Cause Mortality

**Figure 4:**
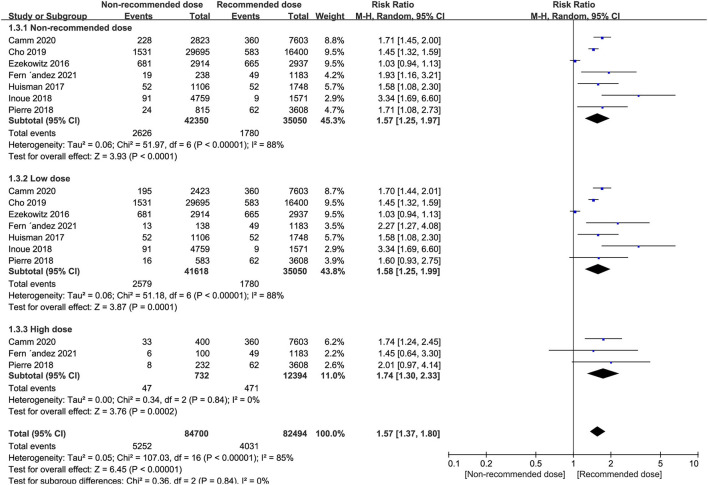
Forest plot for all-cause mortality in non-recommended dose compared to recommended dose of NOACs. NOACs, new oral anticoagulants.

Patients receiving non-recommended dose of NOACs were at significantly higher risk of all-cause mortality than standard dose (RR = 1.57, 95% CI 1.25–1.97, *P* < 0.0001), with similar outcomes evident in low dose group patients (RR = 1.58, 95% CI 1.25–1.99, *P* = 0.0001) and high dose group patients (RR = 1.74, 95% CI 1.30–2.33, *P* = 0.0002) (Figure 4).

Subgroup analyses revealed that the risk of all-cause mortality in patients receiving low dose of dabigatran, rivaroxaban or apixaban, was significantly higher than the recommended dose (RR = 1.47, 95% CI 1.05–2.06, *P* = 0.02 for dabigatran; RR = 1.51, 95% CI 1.05–2.17, *P* = 0.03 for rivaroxaban, and RR = 2.20, 95% CI 1.81–2.67, *P* < 0.0001 for apixaban). However, low dose edoxaban produced a similar risk of all-cause mortality in patients to the recommended dose (RR = 0.95, 95% CI 0.87–1.05, *P* = 0.31). Nevertheless, the all-cause mortality events patients were increased in patients receiving high dose rivaroxaban (RR = 1.72, 95% CI 1.00–2.97, *P* = 0.05), albeit at marginal significance levels (Table 8).

**Table 8:** All-cause mortality of low/high dose of individual NOACs.

**Low dose vs. RD**	**No. of studies**	**No. of participants**	**P for heterogeneity**	***I*^2^** **(%)**	**RR (95% CI)**	**P for test**
Dabigatran	4	27,628	0.0001	86%	1.47 (1.05, 2.06)	0.02^*^
Rivaroxaban	3	26,512	0.09	58%	1.51 (1.05, 2.17)	0.03^*^
Apixaban	1	12,502	–	–	2.20 (1.81, 2.67)	<0.0001
Edoxaban	1	14,014	–	–	0.95 (0.87, 1.05)	0.31
**High dose vs. RD**	**No. of studies**	**No. of participants**	**P for heterogeneity**	***I*^2^** **(%)**	**RR (95% CI)**	**P for test**
Rivaroxaban	2	5,123	0.56	0%	1.72 (1.00, 2.97)	0.05

* *Random-effect models. RD, recommended dose; CI, confidence interval*.

### Cardiovascular Cause of Death

**Figure 5:**
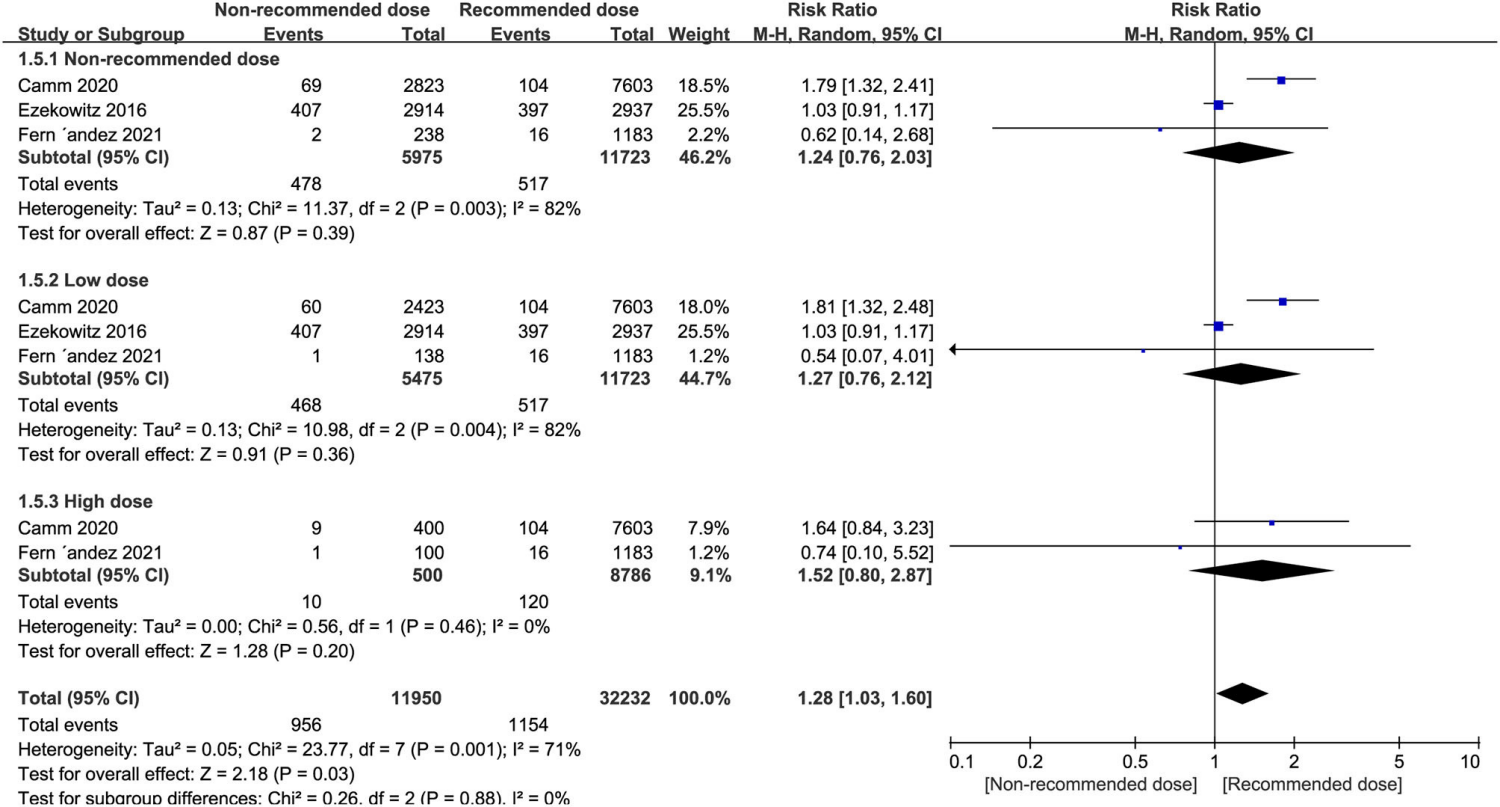
Forest plot for cardiovascular cause of death in non-recommended dose compared to recommended dose of NOACs. NOACs, new oral anticoagulants.

Patients treated with non-recommended dose of NOACs were not more likely to experience a higher risk of cardiovascular death compared to patients receiving standard dose (RR = 1.24, 95% CI 0.76–2.03, *P* = 0.39), and this result was consistent when the non-recommended dose patients were divided into low and high dose groups (RR = 1.27, 95% CI 0.76–2.12, *P* = 0.36; RR = 1.52, 95% CI 0.80–2.87, *P* = 0.20, respectively) (Figure 5).

### Publication Bias and Sensitivity Analysis

Based on a visual inspection of funnel plots (Supplementary Figures 1–4), there was no obvious evidence of publication bias for the selected studies for either reporting efficacy or safety outcomes. But performance bias in these studies inevitably existed. Random effects model showed similar results to the fixed-effects model. AF patients receiving low dose dabigatran, showed high major or clinically relevant bleeding risk in the sensitivity analysis after excluding Ezekowitz 2016 (*I*^2^ = 0%, RR = 1.46, 95% CI 1.22–1.75, *P* < 0.0001). The study of Ezekowitz 2016 (18) had the longest follow-up of 4.6 years, which completed RE-LY trial and continued into RELYABLE study (1, 21), showed low dose of dabigatran was associated with reduced bleeding risk (RR = 0.83,95% CI 0.74–0.91), suggesting that use of low dose dabigatran may reduce long term bleeding risk (Supplementary Figure 8). With regard to all-cause mortality, the pooled results remained stable after sensitivity analysis.

## Discussion

In routine clinical practice, a balance is struck between the risks of stroke and bleeding in AF patients, highlighting the need to optimize interventions using NOACs. Unscheduled dosages of NOACs are often prescribed under real-world conditions, where physicians make judicious decisions according to the patient's age, weight, renal function amongst other performance indicators. Our meta-analysis aimed to investigate the efficacy and safety of using non-recommended dose of NOACs in AF patients. Foremost, our pooled results confirmed that patients receiving scheduled dose of NOACs, no matter dose lower or higher than recommended, had increased risks of stroke/systemic embolism and all-cause mortality. In contrast, there were no significant differences in the risk of bleeding as well as cardiovascular-related death events associated with non-recommended dose of NOACs.

Currently few reported studies have objectively evaluated clinical outcomes in AF patients receiving non-recommended dose of NOACs. In Denmark, Staerk et al. reported that AF patients treated with low dose of NOACs regimens might have a higher risk of stroke and bleeding compared to the recommended dose (22). The ORBIT-AF study showed improved safety outcomes in AF patients with renal indications for NOACs dose reduction where major bleeding outcomes were reduced although there were no significant differences encountered for stroke risk relative to patients receiving recommended dose (23). Yu et al. also suggested that Asian AF patients treated with higher dose of NOACs were at increased risk of stroke or systemic embolism, major bleeding, and all-cause mortality compared with recommended regimens (24).

As shown in our meta-analysis, we found that treating patients with high dose of NOACs produced a trend of higher incidence of stroke or systemic embolism compared with AF patients under standard treatment. This difference may be explained considering the reason underlying the administration of higher dose was likely the associated ischemic comorbidities of these patients. Non-recommended (low or high) dose of NOACs were also significantly associated with a higher risk of death along with composite endpoints (stroke/systemic embolism, major bleeding and death) compared to patients receiving recommended dose. However, after adjustment for baseline characteristics, the composite outcomes, including death risk, were no longer significant, suggested that the higher rates of events associated with non-recommended dose were probably related to the underlying diseases rather than the dose itself (5).

As part of our meta-analysis, we considered the effects of the individual agents as subgroup analyses. We found that patients treated with low dose dabigatran had higher associated risks of stroke/systemic embolism, but nevertheless, exhibited equivalent safety, compared with recommended dose. Consistently, Beyer-Westendorf et al. showed that low dose dabigatran produced increased risks of bleeding and stroke compared with AF patients receiving 150 mg bid (25).

Regarding rivaroxaban, we found patients who received non-recommended dosing showed comparable efficacy to standard regimens, no matter low or high. Nevertheless, low dose rivaroxaban was associated with higher bleeding risk in patients, whereas the higher dose group showed equivalent safety compared to the recommended dose. This contrasts with the findings of the XAPASS study where the incidence rate of stroke/systemic embolism was significantly higher in patients who received low dose rivaroxaban. Moreover, after adjustment for baseline characteristics, the authors reported that the rates of major bleeding were similar in for patients receiving either low or recommended dose of rivaroxaban (26). Other comparisons can be made with the EXPAND and SAKURA AF registry studies where the incidence rates of stroke/systemic embolism and major bleeding were both comparable between rivaroxaban interventions using low and recommended dose (6, 27).

Our subgroup analysis of apixaban-treated patients showed lower dose were associated with significantly higher risk of stroke and systemic embolism, major or clinically relevant bleeding compared to patients receiving the recommended dose. Our data are partly consistent with a prior study showing that low dose apixaban in patients with no renal indication for dose reduction was associated with significantly higher risk of stroke but without a reduction of major bleeding compared to patients taken standard dose of apixaban (23).

Lastly, the risk of stroke or systemic embolism in AF patients receiving low dose edoxaban was higher than patients treated with the recommended dose, but nonetheless this regimen had a superior safety outcome. This notion is supported by a published study reporting that the rate of ischemic stroke was higher with a low dose edoxaban regimen, but with greater risk reduction in bleeding compared to warfarin (28).

In summary, our pooled meta-analysis showed low dose of NOACs had worse efficacy for stroke/systemic embolism prevention, but without reducing in major bleeding, and with increased all-cause mortality compared to standard use. Moreover, high dose of NOACs did not show better outcomes in stroke/systemic embolism prevention in AF patients. Therefore, non-recommended dose of NOACs should be carefully prescribed since off-label use of any NOAC may cause different clinical outcomes. Furthermore, there may be subtle differences among the individual NOACs, such as pharmacologic properties, frequency of use, dose, interaction between drugs or other currently undefined aspects to consider. So, direct comparative studies are warranted to determine whether these are real differences in clinical efficacy and safety outcomes between individual NOACs.

## Limitations

Some limitations should be acknowledged of this meta-analysis. First, pooled data were collected from different trials with differences in their design, including baseline participant characteristics, agents and dose regimens, definitions of efficacy and safety outcomes, along with differences in the length of follow-up time. Any combination of these variables could account for the source of moderate to high degree of statistically heterogeneity in our meta-analysis. This point needs further validation and it is also important to consider that heterogeneity can occur by chance and would almost certainly be found with meta-analysis involving many large studies (29). Furthermore, most of the studies included in our meta-analysis were non-randomized observational studies and were limited by potential selection and ascertainment bias. Moreover, the efficacy or safety outcomes in some of the included studies were not fully adjusted with medical records or baseline characteristics. Therefore, clinical events might be associated with off-label use but not causally related to dosing, thus introducing a possible risk of bias and misclassification of the end points (12), indicating the need for caution in interpreting outcomes.

## Conclusions

Based on the available data, our study concludes that AF patients treated with non-recommended dose of NOACs may be at higher risk of stroke/systemic embolism and all-cause mortality compared with recommended dose, albeit the benefit of without reducing in major bleeding. Of particular interest, dabigatran, rivaroxaban, apixaban, edoxaban all differed in their efficacy and safety outcomes with non-recommended dose regimens.

## Data Availability Statement

The original contributions presented in the study are included in the article/Supplementary Material, further inquiries can be directed to the corresponding author/s.

## Author Contributions

Data analysis, interpretation, and manuscript writing were performed by XK. Literature search, study selection, data extraction, and quality assessment were performed by XK, LP, YZ, SM, LZ, and WZ. YZ and HL were responsible for the conception and design of the study. YZ and HL revised the manuscript carefully. All authors contributed to the article and approved the submitted version.

## Funding

This work was supported by the grant from Beijing Lab for Cardiovascular Precision Medicine, Beijing, China (PXM2020_014226_000017_00377132_FCG).

## Conflict of Interest

The authors declare that the research was conducted in the absence of any commercial or financial relationships that could be construed as a potential conflict of interest.

## Publisher's Note

All claims expressed in this article are solely those of the authors and do not necessarily represent those of their affiliated organizations, or those of the publisher, the editors and the reviewers. Any product that may be evaluated in this article, or claim that may be made by its manufacturer, is not guaranteed or endorsed by the publisher.

## Abbreviations

AF: atrial fibrillation
NOACs: new oral anticoagulants
VKA: vitamin K antagonists
EMA: European Medicines Agency
FDA: U.S. Food and Drug Administration
PMDA: Japanese Pharmaceuticals and Medical Devices Agency
BMI: body mass index
CrCl: creatinine clearance
PRISMA: Preferred Reporting Items for Systematic Reviews and Meta-Analysis statement
NOS: Newcastle-Ottawa Scale
SE: systemic embolism
ISTH: International Society on Thrombosis and Hemostasis
RR: risk ratios
OC: observational cohorts
RCT: randomized controlled trials
NA: not available
MI: myocardial infarction
TIA: transient ischemic attack
CNS: central nervous system
CV: cardiovascular
GI: gastrointestinal
MACE: major adverse cardiovascular events
M ± SD: mean ± standard deviation
IQR: interquartile range
RD: recommended dose
LD: low dose
HD: high dose
CI: confidence interval.
